# Prescribing behavior of antidepressants for depressive disorders: A systematic review

**DOI:** 10.3389/fpsyt.2022.918040

**Published:** 2022-09-09

**Authors:** Mary K. Lam, Lawrence T. Lam, Kerryn Butler-Henderson, Jonathan King, Tahnee Clark, Peta Slocombe, Katherine Dimarco, Wendell Cockshaw

**Affiliations:** ^1^School of Health and Biomedical Sciences, RMIT University, Melbourne, VIC, Australia; ^2^Faculty of Medicine, Macau University of Science and Technology, Macau, Macau SAR, China; ^3^Specialty of Child and Adolescent Health, The University of Sydney, Sydney, NSW, Australia; ^4^Faculty of Health, University of Technology Sydney, Sydney, NSW, Australia; ^5^Lysn (A Danewell Health Company), Sydney, NSW, Australia; ^6^Sydney Medical School, The University of Sydney, Sydney, NSW, Australia

**Keywords:** antidepressants, depressive disorder, systematic review, prescribing behavior, psychopharmacotherapy

## Abstract

**Objective:**

Guidelines for the prescription of antidepressants for Depressive Disorders (DD) have been in place for a long time. However, there is a lack of systematic information on the prescribing behavior of antidepressants in evidence-based clinical practice in psychopharmacotherapy of depressive disorders. This may suggest a lack of implementation of clinical guidelines by clinicians. Existing literature mainly focuses on specific issues or medications. To provide general information on the prescribing behavior of antidepressants for depressive disorders, a systematic review of available studies since 2013 was conducted.

**Methods and materials:**

To ensure a structured and systematic approach for the literature search and subsequent review process, the PRISMA guidelines for systematic reviews were followed. Major medical and health and psychological databases were used for the literature search. These included Ebsco Host, OVID, PubMed, Science Direct, Scopus, and Web of Science. The online application “Covidence” was employed to manage the titles collected and the full articles retrieved from the initial literature search. Upon finalizing the list of selected studies, data extraction was then conducted using a build-in function of the Covidence platform with the required information pre-set on a template for data extraction. The extracted information was tabulated and summarized in a table.

**Results:**

Forty-one studies were identified after an extensive search of the literature following the PRISMA guidelines. Of these, 37 quantitative studies providing useful information were systematically reviewed and information extracted. There was a high level of heterogeneity among these studies with different foci or characteristics. Most studies were conducted in or utilized data obtained from hospital and primary healthcare settings. SSRIs were the most commonly prescribed type of antidepressant in the past decade, particularly among younger patients. Among these studies, antidepressants were mainly prescribed by psychiatrists with some by other physicians and general practitioners. This might reflect differences in legislation regarding professional requirements for prescribers or clinical practices.

**Conclusions:**

A few themes that would be considered important in terms of the effect of prescription behavior on depression, specifically children/adolescents, special target populations, and off-label prescription. The results highlighted the need for more studies on a community-based approach and the role of GPs in the treatment of DD.

## Introduction

In clinical practices, prescriptions of medications must be conducted according to clinical guidelines. The guidelines for the prescription of antidepressants for Depressive Disorders (DD), which include Major Depressive Disorder (MDD), Dysthymia, and Premenstrual Disorders, have been in place for a long time. In a review of guidelines published between 2007 and 2012, Wang et al. found that of the available guidelines reviewed four were developed in the USA, one by an international group, and one in Korea (1). Upon comparing these guidelines, Wang et al. concluded there were many similarities in the recommendation of DD treatment options for the initial treatment, second step treatment in case of non-response and/or incomplete response to initial treatment, and third step treatment if patients are non-responsive to second step treatment (1). Mono-pharmacotherapy antidepressants are preferred as the first-line treatment for initial treatment of DD without psychosis, and for mild to moderate DD. Selective Serotonin Reuptake Inhibitors (SSRIs) and Serotonin Norepinephrine Reuptake Inhibitors (SNRI) are the preferred antidepressants during initial treatment, with some exceptions. For second-line treatment, a combination of different antidepressants is used (1).

However, there is little systematic information on many aspects of the prescribing behavior of antidepressants from evidence-based clinical practice in psycho-pharmacotherapy of depressive disorders. There is little information regarding prescriptions for first-line treatment and subsequent treatments, the types of antidepressants commonly prescribed as monotherapy and polypharmacotherapy, the setting in which antidepressants are prescribed, and prescription patterns in different populations. To ascertain any similar and relevant existing systematic reviews on the same topic, a search of the main health-related databases was conducted prior to the commencement of the current review study. The literature scan concentrated on reviews published in the past 5 years. Results suggest the existence of previous systematic reviews in the literature on the topic of antidepressant prescribing behavior (2–9). However, most of these reviews focused on a specific antidepressant usage or a targeted patient group. For example, Swainason et al. (3) focused on the usage of ketamine in adults with MDD, and Ostuzzi et al. (4) studied antidepressants use for patients with cancer.

Few studies provide a broader and more general review of the prescribing behavior of clinicians including psychiatrists, physicians, and other health professionals. This review aims to fill this knowledge gap and provide general information on the prescribing behavior of antidepressants for depressive disorders without other co-morbidities, such as anxiety disorders, psychosis, and substance use disorders. The reason for a focus on depressive disorders only is to allow clinicians to have a fundamental view of the current prescription pattern for a major mental health issue before venturing into more complex problems.

## Methods

To ensure a structured and systematic approach for the literature search and subsequent review process, the PRISMA guidelines for systematic reviews were followed. Major medical and health and psychological databases were used for the literature search. These included Ebsco Host, OVID, PubMed, Science Direct, Scopus, and Web of Science with the following search terms and combinations: (prescri^*^) AND (medicat^*^ OR antidepressant) AND (behavior OR behavior OR pattern) AND (depressive OR depression). Some restrictions were imposed to ensure the studies included were peer-reviewed journal articles; written in English, were original studies, and published since 2013. A restriction was imposed on the year of publication due to the global change in clinical guidelines and practices implemented in 2012.

The online application “Covidence” was employed to manage the titles collected and the full articles retrieved from the initial literature search. The following steps were undertaken to ensure that studies selected for final data extraction satisfied all selection criteria. First, abstracts were screened and analyzed for the key topic of the study, namely prescribing behavior of antidepressants for a major depressive disorder either as the primary or a co-morbidity diagnosis. This was conducted by authors ML, LL, KBH, WC. Second, full texts of the potentially acceptable articles were examined to assess their suitability for final data extraction. This step was conducted by three authors, ML LL and KBH independently based on the selection criteria. At the end of the independent selection, the results of selection by the authors were matched for any discrepancies. Differences were discussed and discrepancies were resolved through comparison to the selection criteria.

The following criteria were applied to the selection of studies: (1) Publications with a proper study design and prescribing behavior identified; (2) Studies that provided clear descriptions allowing an accurate assessment of whether key information on important variables could be extracted; (3) Studies that included information on variables including the year of publication, country, prescribers, the target population, sample size, setting of prescription, types, and names of antidepressants, reasons for a prescription; and (4) Studies published in English. Studies that focused on the knowledge, attitudes, and perceptions of clinicians toward the prescription of antidepressants were excluded from this review.

**Figure 1:**
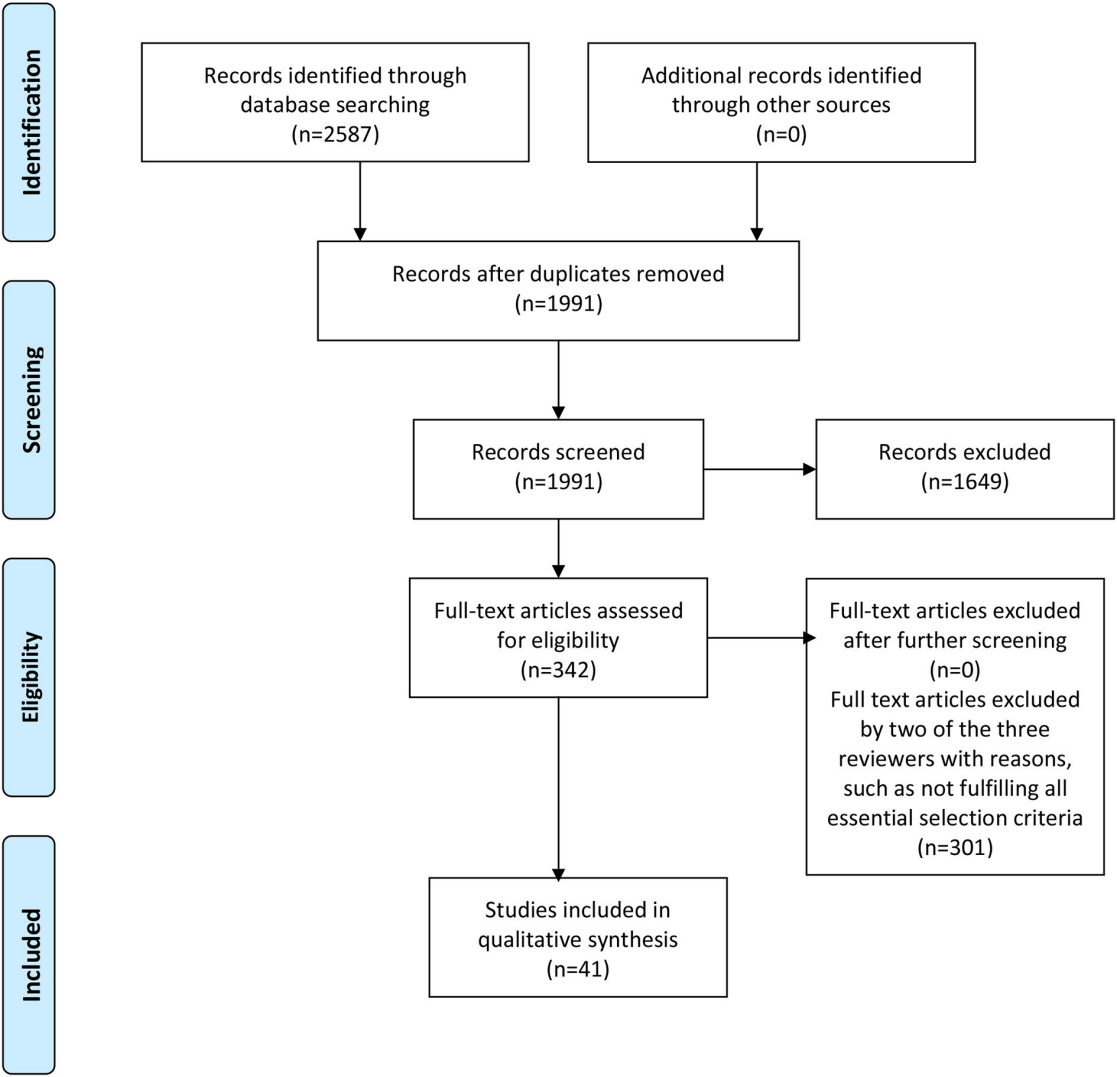
PRISMA flow chart.

Upon finalizing the list of selected studies, data extraction was then conducted using a build-in function of the Covidence platform with the required information pre-set on a template for data extraction. The extracted information was tabulated and summarized in a table. The full articles were reviewed thoroughly to elucidate the main characteristics or themes of each study. These themes were also tabulated and presented in Table 1. Due to the observational nature of these studies, a descriptive approach was used to comment on any potential biases and/or limitations of the studies. The systematic literature searches and review process was summarized schematically in Figure 1 following the PRISMA chart format (47).

**Table 1:** Summary of article characteristics.

**References**	**Country**	**Method**	**Participants**	**Theme**
**Quantitative studies**				
Ahmad et al. (10)	India	Review of medical records	156	Elderly and simple descriptive study
Ghosh and Roychaudhury (11)	India	Prospective case series study with a convenience sample method.	510	Adults and polypharmacotherapy
Ball et al. (12)	USA	A Secondary data analysis study	5,012	All ages and second-line treatment
Al Za'abi et al. (13)	Oman	Review of medical records	NA	Adults and simple descriptive study
Zhang et al. (14)	China	A Secondary data analysis study	8,484	Adults and change of prescription
Tripathi et al. (15)	India	A cross-sectional study	312	Adults
Reardon and Creado (16)	USA	A cross-sectional study	NA	Adults and special patient group-athletes
Jacob and Kostev (17)	Germany	A Secondary data analysis study	89,962	Adults and no gender difference
Dold et al. (18)	Mult-European countries	A cross-sectional study	1,181	Adults and polypharmacotherapy
Chee et al. (19)	REAP project	A cross-sectional study	NA	Children/Adolescents, off-label prescription, and AD prescribed not for depression
Chattar et al. (20)	India	A cross-sectional study	284	Adults and change of prescription
Treviño et al. (21)	USA	A secondary data analysis study	54,107	Adults and correct dosage
McIntyre et al. (22)	USA	A secondary data analysis study	130,626	Adults and treatment for sub-type of MDD
Massamba et al. (23)	Canada	A longitudinal study	263	Elderly and adequate treatment
Chon et al. (24)	Korea	Review of medical records	2,190	Children/adolescents and off-label prescription
Bose et al. (25)	India	A cross-sectional study	200	Adults and off-label prescription
Dharni and Coates (26)	Australia	Review of medical records	189	Children/adolescents and off-label prescription
Zhong et al. (27)	REAP project	A Secondary data analysis study	671	Elderly and survey of psychiatrists
Gers et al. (28)	Belgium	Observational case series study	239	Elderly and incorrect indication for prescription
Fata Nahas and Syed Sulaiman (29)	Malaysia	Observational case series study	119	Adults and change of prescription pattern overtime
Bandoli et al. (30)	USA	A Secondary data analysis study	162	Adults and special patient group—pregnant women
Verhaak et al. (31)	The Netherlands	A secondary data analysis study	326,025	Adults, Long-term use, and bias in prescription
Vadiei and Bhattacharjee (32)	USA	A secondary data analysis study	262	Adults, special patient group- with kidney diseases
Saito et al. (33)	Japan	A cross-sectional study	6,080	Children/adolescents, reasons for prescription
Tayem et al. (34)	Bahrain	A cross-sectional study	226	All ages and simple descriptive study
Lunghi et al. (35)	Italy	Retrospective cohort study	18,307	All ages and long-term use
Lukmanji et al. (36)	Canada	A secondary data analysis study	NA	Children/adolescent and trend of prescription
Heald et al. (37)	UK	A secondary data analysis study	NA	All ages and change of prescription
Hadia et al. (38)	India	Observational case series study	37	Adults and drug use problem
Chen et al. (39)	Taiwan	A longitudinal study	105	Adults and patient classification based on prescription pattern
O'Neill et al. (40)	Ireland	A longitudinal study	817	Elderly, long-term use, and bias in prescription
Mössinger and Kostev (41)	Germany	A secondary data analysis study	138,097	Adults and age effect
Kamran et al. (42)	Pakistan	Observational case series study	302	Adults and polypharmacotherapy
Hung et al. (43)	Taiwan	A longitudinal study	97	Adults and first-line and second-line treatment
Hattab et al. (44)	Palestine	A secondary data analysis study	159	Adults and polypharmacotherapy
Hashimoto et al. (45)	Japan	Observational case series study	1,238	Adults and polypharmacotherapy
Hansen et al. (46)	Norway	A secondary data analysis study	49,967	Adults and bias in prescription

REAP project = 10 Asian/region countries including China, Hong Kong, India, Indonesia, Japan, South Korea, Malaysia, Singapore, Taiwan, and Thailand.

## Results

In total, 41 studies were retained and reviewed after the systematic search and screening process. Of these, two were qualitative studies and were reviewed separately using qualitative review methodologies. Another two studies were surveys of clinicians regarding their choices or preferences of drug use and their perception of the drugs, and not about their prescribing behavior, leaving 37 studies for further review.

Information was extracted from the 37 quantitative studies using a systematic approach following the PRISMA guidelines. The information was tabulated in Table 1 with Supplementary materials included in the Appendix. Of these 37 studies, slightly more than half (*n* = 22, 56%) were published recently between 2018 and 2021 with slightly less than half (*n* = 16, 43%) of the studies containing data from Asian countries, followed by European countries (*n* = 8, 22%), and the USA (*n* = 6, 16%). The most common type of study design was secondary data analysis (*n* = 14, 38%), followed by cross-sectional studies (*n* = 8, 22%), and patient case-series studies (*n* = 6, 16%). The majority (*n* = 32, 86%) provided the number of patients included in the studies with a total of 836,386 patients (mean = 2,6137.1, s.d. = 65,884.1, min = 37, max = 326,025), with most studies on adults only (*n* = 21, 57%) and followed equally by all ages, children and adolescents, and elder ages (*n* = 5, 14%). One study was on pregnant women, another was among athletes, and the last was a survey of psychiatrists.

In most of the studies (*n* = 29, 78%) depression was reported as the primary diagnosis, three (*n* = 3, 8%) reported as co-morbidity of other illnesses, and the rest did not report any details on the specific depression diagnosis, such as unipolar depression, bipolar depression, or dysthymia. About half (*n* = 19, 51%) of these studies were set in hospitals including both inpatients and outpatients of the public and private sectors, seven (*n* = 7, 19%) in primary care, five (*n* = 5, 14%) in multiple settings, and the rest in other settings including a specialized sports psychiatric center and patients involved in a large project involving 10 Asian countries. In terms of the prescriber of antidepressants, about half (*n* = 18, 49%) of the studies reported prescriptions by psychiatrists only, six (*n* = 6, 16%) involved GPs and other physicians, another six involved a mix of psychiatrists, GPs, and physicians, one reported other physicians, another recorded psychologist, and the rest were without a clear indication of the prescribers. This might reflect some differences in legislation regarding professional requirements for prescribers or clinical practices. No specific trends were observed among these studies regarding the change of prescribers from psychiatrists to general practitioners or a change of drug type prescribed. Among these only 6 (16%) reported the number of prescribers with a total of 1,515 (mean = 252,5, s.d. = 220.8, min-40, max = 652). For the type of antidepressants, the majority (*n* = 33, 89%) of the studies reported that SSRIs were prescribed, in 29 (78%) TCAs were mentioned, and 21 (57%) SNRIs were recorded. Tetracyclic Antidepressants (TeCAs) were indicated in 14 (38%) studies and Serotonin antagonist and reuptake inhibitors (SARIs) in 11 (30%). Monoamine oxidase inhibitors (MOAIs) only occurred in 3 (8%) studies and 1 study reported the use of alternative and traditional medicine (ATM).

It was noted that there was a high level of heterogeneity among these studies with different foci or characteristics. Hence, these characteristics were also extracted and summarized as themes of each study. These themes were presented in Table 1. As shown, some common themes were derived from these studies, such as specific age groups, special target populations, off-label prescription, biases in prescription, and polypharmacotherapy. Further analyzes were conducted on a few themes that would be considered important in terms of the effect of prescription behavior on depression, specifically children/adolescents, special target populations, and off-label prescription. The results were summarized below.

### Children/adolescents

Five studies focused on the prescription of antidepressants for children and adolescents (19, 24, 26, 33, 36). Of these five studies, three were conducted in Asian countries (19, 24, 33), and one in Australia (26), and another in Canada (36). Nearly all studies were conducted in hospital and primary care settings, except for one in community care (26). Prescribers were mainly psychiatrists and physicians. Most studies indicated that the majority of patients were treated with mono-pharmacotherapy at a level of about 92–95% (19, 24). Multiple types of antidepressants were used for young patients with SSRIs as the most common type. Three studies also focused on the off-label use of antidepressants among young people with depression (19, 24, 26). Based on these studies, off-label prescription of antidepressants for children and adolescents was common in many countries. This was also a theme that will be further reported below.

### Special target populations

In terms of different patient populations, apart from the elderly and children/adolescents, some studies concentrated on special target populations. These include the Reardon and Creado (16) study on athletes, another study by Bandoli et al. (30) on pregnant women, and the study among patients with chronic kidney disease by Vadiei abd Bhattacharjee (32). All three studies were conducted in the US. In the survey study on the prescribing practice of sports psychiatrists who were members of the International Society of Sports Psychiatry, it was found that bupropion was the top choice of medication for depression in young athletes (16). Several reasons were provided for the choice of medications including increased energy, lower tendency to cause sedation, weight gain, cardiac side effects, and tremors (16). In the Bandoli et al. study, 166 pregnant women, who had been prescribed an antidepressant between 91 days prior to the last menstrual period and 32 weeks of gestation, were followed longitudinally (30). The most common antidepressant prescribed was SSRIs (78%), followed by bupropion (14%) and tricyclics (8%), with four groups identified based on the median cumulative fluoxetine equivalent dosage (30). It was also found that the highest usage group during pregnancy was associated with reduced birth weight. However, this was a small study with only 162 patients. In a study on the prescription of antidepressants for patients with chronic kidney disease, Vadiei et al. found that half of these patients received treatment for depression. The main setting of treatment for depression was in primary care with the most prescribed antidepressant SSRIs accounting for about 35%, followed by bupropion and mirtazapine (7%) (32).

### Off-label prescription

Four studies examined off-label prescriptions for depression that the usage or administration of specific types of medication was not included in the product information or not yet approved for the users' population. As aforementioned, the target population of three of these studies was children and adolescents, and adults (19, 24–26). Two of these studies were conducted in Asian countries, one in India and one in Australia, mostly in a hospital or primary care setting with only one in the community (26). Most young patients were treated with mono-pharmacotherapy, with off-label prescriptions found to be a common practice, particularly in young patients. In the study by Chee et al. almost a third (30%) of antidepressant prescriptions were for diagnoses other than depression and anxiety, while the reasons and diagnoses for off-label prescriptions were not provided in other studies (19). Chon et al. reported that escitalopram and fluoxetine were the two antidepressants approved for the treatment of depression in children and adolescents by the FDA accounting for 32 and 24% of total patients respectively. The authors concluded that a large proportion of prescriptions for depression in Korean youths were off-label (24). Dharni and Coates studied the prescription pattern of antidepressants among young people in a community mental health center in Australia and concluded that about half of young people were treated with a single antidepressant (51%), with SSRIs the most common type. These antidepressants were mostly used off-label (26). In the study by Bose et al., among Indian adults in a tertiary hospital setting, it was found that 43% of antidepressant prescriptions were used for an off-label indication and fluoxetine (44%) and escitalopram (42%) were the most frequently prescribed in that manner (25). It was also found that the off-label indications were somatoform disorder (12.5%) and followed by generalized anxiety disorder (8.5%).

In terms of the study design, the majority were reviews of medical records, secondary data analysis, and cross-sectional studies. Few adopted a study design that would provide evidence with greater strength in terms of evidence-based practice. Of the 37, four were longitudinal studies that follow patients for some time (23, 39, 40, 43). These studies would be considered more pertinent in terms of the information provided by the patients. Another study worth noting was the large Italian retrospective cohort study of 18,307 patients of all ages by Lunghi et al. (35). This study investigated the long-term prescription patterns and provided detailed information on the changes in these prescriptions.

For some of the topics mentioned in the introduction, such as prescriptions for first-line treatment and subsequent treatments, little information was provided by these studies. This also applied to the types of antidepressants commonly prescribed as monotherapy and polypharmacotherapy.

## Discussion and conclusion

This study aims to investigate the prescription behavior of antidepressants for depressive disorders by reviewing recently published reports. Of interest in this review is the systematically generated information on different aspects of the prescribing behavior of antidepressants with special attention to some prescribing patterns that may impact the health outcomes of depressed patients. A few outstanding observations have resulted from this review. First, most of the reviewed studies were conducted in, or utilized, data obtained from hospital and primary healthcare settings. Only one study was carried out in a community mental health center. Second, the past decade has seen SSRIs used as a commonly prescribed type of antidepressant, particularly among younger patients. However, there is a more recent trend to prescribe SNRIs and miscellaneous types of antidepressants, such as bupropion and mirtazapine. Third, most of these studies are focused on prescriptions of antidepressants by psychiatrists and other physicians. Not many studies involved GPs and other health professionals. Fourth, due to the high level of heterogeneity in the reviewed studies in terms of objectives, only a few common themes emerged.

The results obtained from this review provide some insights into the current status of antidepressant prescriptions for the treatment of DD. The very fact that only one study focused on the community mental health setting is revealing. This reflects a lack of attention on community-based pharmacotherapy treatment in terms of appropriate and effective treatment options for DD. There may be many reasons for such a lack of attention. One reason is that most patients with DD receiving community-based treatment are mainly in a sub-acute phase of the disorder. Pharmacotherapy treatment may mainly focus on the maintenance of the medication for treatment stabilization. Another reason may be due to the fact that other treatment options or approaches are available for DD in a community setting, such as counseling or cognitive behavioral therapy, in conjunction with other therapeutic approaches. These treatment options may be provided in a complementary or supplementary manner by specialized health professionals such as the Community Mental Health Team (CMHT). As a result, pharmacotherapy may play a slightly different role in the treatment of DD in comparison to patients in the acute phase of the disorder. Another point worth noting is the lack of studies on GPs and other healthcare professionals as prescribers of antidepressants. This may be because most of these studies have been conducted by psychiatrists and thus demonstrate a bias toward their profession. On the other hand, this may also reflect a lack of attention to GPs and other healthcare professionals as an important resources for providing treatment and services for people with mental health problems in the community.

When comparing the results obtained from this review with the current clinical guidelines on the antidepressant prescription provided by professional bodies, such as the Royal Australian and New Zealand College of Psychiatrists (RANZCP) Mood Disorder Guidelines 2020 (48) and the National Institute for Health and Care Excellence (NICE) Guidelines Depression in adults: treatment and management 2022 (49), some observations are noted. RANZCP does not outline specific antidepressant classes for first, second, and third-line treatment. The guidelines recommend seven antidepressants from seven classes (escitalopram, vortioxetine, agomelatine, venlafaxine, mirtazapine, bupropion, amitriptyline). The choice of antidepressant is based on clinical judgement patient factors, efficacy and tolerability. Based on this, the RANZCP guidelines are not reflected in the study findings that showed high weighting toward the prescription of SSRIs, SNRIs and TCAs. If the RANZCP guidelines were followed, a wider spread across antidepressant classes may be seen. However, clinicians, particularly in primary care, may be most familiar with SSRIs and SNRIs and continue to predominantly prescribe those. The study covered a range of countries, and there can be local variation in antidepressant availability. Not all medications on the RANZCP list may be available in all countries. On the other hand, NICE recommends that “less severe depression” be treated with SSRIs, and “more severe depression” treated with SSRIs or SNRIs, or another antidepressant if indicated based on previous clinical treatment history. NICE recommend that TCAs and MAOIs be used in secondary care settings. The study findings showed concordance with the NICE guidelines, in that SSRIs and SNRIs were very commonly prescribed. TCAs were prescribed more than expected based on the NICE guidelines, which commented on their toxicity in overdose, and recommended prescription of these medications in specialized settings.

There are strengths and limitations in this systematic review as in all others. This review study followed the PRISMA guidelines for systematic reviews ensuring the quality of the systematic review. Reviewers have observed the selection criteria for the inclusion of studies in the review with a dual process of article selection and data extraction. The use of the Covidence online platform has aided in implementing standardized procedures. In terms of limitations pertaining to individual studies, comments were made in the information extraction table. However, some general limitations have been identified. First, due to the heterogeneity of these studies with different objectives, information is missing on some variables, such as reasons for prescription and the average dosage prescribed. Second, most studies were conducted in a hospital or primary care setting by psychiatrists. Moreover, there is an uneven distribution of geographical origin of these studies with nearly half of the studies being conducted in Asian countries. Hence, this may constitute a publication bias. Finally, due to the heterogeneity of these studies, very few common themes could be derived, thus only the more obvious themes were discussed.

## Author contributions

ML: study design, title and abstract screen, full text screen, data extraction, and write manuscript. LL: full text screen, data extraction, and write manuscript. KB-H: study design, database search and upload papers to Covidence, title and abstract screen, and full text screen. JK, TC, PS, and KD: comment and edit manuscript. WC: study design and title and abstract screen. All authors contributed to the article and approved the submitted version.

## Funding

This review study has received funding support from Lysn.

## Conflict of interest

This research is sponsored by Lysn Pty Ltd (an online mental health service) to find out clinicians' prescribing behavior of antidepressants for depressive disorders. Authors JK, TC, PS, and KD are employed by Lysn Pty Ltd. The remaining authors declare that the research was conducted in the absence of any commercial or financial relationships that could be construed as a potential conflict of interest.

## Publisher's note

All claims expressed in this article are solely those of the authors and do not necessarily represent those of their affiliated organizations, or those of the publisher, the editors and the reviewers. Any product that may be evaluated in this article, or claim that may be made by its manufacturer, is not guaranteed or endorsed by the publisher.
