# Supplementary material for: Prescribing behavior of antidepressants for depressive disorders: A systematic review
Source: Front Psychiatry. 2022 Sep 9;13:918040. doi: 10.3389/fpsyt.2022.918040 (PMC9501861; doi:10.3389/fpsyt.2022.918040)
Supplement: Supplementary file 1 [file Data_Sheet_1.docx]

Appendix. Supplementary Summary Table

| **First author and year of publication** | **Country of data source** | **How data were collected** | **Study population** | **Setting in which data were collected** | **Number of patients reported** | **Depression was reported as** | **Prescribers** | **Number of prescribers reported** | **Types of AD reported** | **Number of drugs prescribed per episode of treatment reported** | **Length of treatment per episode reported** | **Reasons for drug choice reported** | **Remarks** |
| --- | --- | --- | --- | --- | --- | --- | --- | --- | --- | --- | --- | --- | --- |
| Ahmad et al.,  2013 | India | Review of medical records | Elderly | Public hospitals | 156 | A comorbidity | Psychiatrists | Not reported | *SSRIs*  Escitalopram Fluoxetine Sertraline  *TCAs*  Amitriptyline Dothiepin  *TeCAs*  Mirtazapine | 10 of the 156 were prescribed with a mix of SSRI and Atypical Antidepressant, others were prescribed with a single antidepressant | Not reported | Not reported | Simple descriptive study with a very small sample size |
| Ghosh et al.,  2014 | India | Prospective case series study with a convenience sample method. | Adults | Public hospitals | 510 | A primary diagnosis | Psychiatrists | Not reported | *SSRIs*  Fluoxetine Sertraline  *TCAs*  Amitriptyline | Not reported | Sertraline 20.9 (s.d.=0.9); Amitriptyline 20.8 (s.d.=0.6); Fluoxetine 20.9 (s.d.=0.8) | Not reported | No specific comments |
| Ball et al.,  2014 | USA | A Secondary data analysis study | All ages | Multiple settings | 5,012 | A primary diagnosis | Psychologist and others | Not reported | *SSRIs*  Citalopram Escitalopram Fluoxetine Fluvoxamine Paroxetine Sertraline  *SNRIs*  No details  *TCAs*  Trazodone | Not reported | On average 9.8 (sd 2.3) months | Not reported | Study focused on the second-line Rx after an initial Rx with SSRIs. Approximately a third of patients who are treated with an SSRI will continue with mono-therapy, while the majority of patients will require a second-step intervention. TCAs were most commonly used. |
| AlZa'abi et al., 2014 | Oman | Review of medical records | Adults | Public hospitals | NA only 381 prescriptions for depression | A primary diagnosis | Not reported | Not reported | *SSRIs*  Fluoxetine Paroxetine  *SNRIs*  Duloxetine Venlafaxine  *TCAs*  Imipramine  *TeCAs*  Mirtazapine | Not reported | Not reported | Not reported | A simple descriptive study with a small sample |
| Zhang et al., 2016 | China | A Secondary data analysis study | Adults | Hospital outpatient clinic/centre | 8,484 | A primary diagnosis | Not reported | Not reported | *SSRIs* Citalopram Escitalopram Fluoxetine Fluvoxamine Paroxetine Sertraline *SNRIs*  Duloxetine Venlafaxine  *TCAs*  Trazodone  *TeCAs*  Mirtazapine  *SARIs*  Trazodone  *NaSSA*  No details | Not reported | Not reported | Not reported | SSRIs were prescribed to 60.5% of patients, followed by 9% receiving an NaSSA, 8.3% receiving SNRIs, 2.3% receiving TCAs, and 4.9% receiving other antidepressants |
| Tripathi et al.,  2016 | India | A cross-sectional study | Adults | Hospital outpatient clinic/centre | 312 | A primary diagnosis | Psychiatrist and physicians | Not reported | *SSRIs*  Escitalopram Fluoxetine Fluvoxamine Paroxetine Sertraline *SNRIs*  Desvenlafaxine  Duloxetine Venlafaxine  *TCAs*  Amitriptyline Imipramine Clomipramine  *TeCAs*  Mirtazapine  *SARIs*  Trazodone | Only 9.6% received more than one type of AD | Not reported | Not reported | No specific comments |
| Reardon et al.,  2016 | USA | A cross-sectional study | Athletes presumably young adults | Sports psychiatry setting | Not reported | A primary diagnosis | Psychiatrist | 40 | *SSRIs*  Citalopram Escitalopram Fluoxetine Sertraline  *SNRIs*  Venlafaxine Bupropion | Not reported | Not reported | Not reported | No specific comments |
| Jacob et al.,  2016 | Germany | A Secondary data analysis study | Adults | Any settings | 89,962 | A primary diagnosis | Psychiatrist | 223 | *SSRIs*  No details  *SNRIs*  No details  *TCAs*  No details  *TeCAs*  No details  *ACM*  No details | Not reported | Not reported | Not reported | SSRIs and TCA were the most commonly prescribed AD with 40.1% and 12.4% respectively. |
| Dold et al.,  2016 | Multiple European countries | A cross-sectional study | Adults | Inpatient and outpatient settings | 1,181 | A primary diagnosis | Psychiatrist | Not reported | *SSRIs*  Citalopram Escitalopram Fluoxetine Fluvoxamine Paroxetine Sertraline *SNRIs*  Duloxetine Venlafaxine  Milnacipran  *TCAs*  Amitriptyline Clomipramine  *TeCAs*  Mirtazapine  *NaSSA*  No details | Not reported | Not reported | Not reported | 53.4% of all included MDD patients received  a SSRI, 23.6% SNRI, 8.2% NaSSA,  5.1% TCA  most commonly pre-  scribed individual antidepressant was escitalopram (18.4%) |
| Chee et al.,  2016 | 10 Asian/region countries including China, Hong Kong, India, Indonesia, Japan, South Korea, Malaysia, Singapore, Taiwan, and Thailand. | A cross-sectional study  Survey of psychiatrists of the REAP project | Children/Adolescents | Public and private hospitals and outpatient clinics/centres | Not reported | A primary diagnosis | Psychiatrist | Not reported | *SSRIs*  Citalopram Escitalopram Fluoxetine Fluvoxamine Paroxetine Sertraline *SNRIs*  Duloxetine Venlafaxine  *TCAs*  Amitriptyline  Imipramine Clomipramine  *TeCAs*  Mirtazapine | Not reported in details  only mentioned 5.5% polypharmacy | Not reported | Not reported | No specific comments |
| Chattar et al.,  2016 | India | A cross-sectional study | Adults | Psychiatric outpatient department of a public hospital | 284 | A primary diagnosis | Psychiatrist | Not reported | *SSRIs*  Citalopram Fluoxetine Fluvoxamine Paroxetine Sertraline  *TCAs*  Amitriptyline  Imipramine  *TeCAs*  Mirtazapine | Not reported | Not reported | Not reported | A study with small sample size |
| Treviño et al.,  2017 | USA | A Secondary data analysis study | Adults | Not reported | 54,107 | Not reported | Not reported | Not reported | *SSRIs*  Citalopram Escitalopram Fluoxetine Fluvoxamine Paroxetine Sertraline  Vilazodone *SNRIs*  Duloxetine Venlafaxine  *TCAs*  Amitriptyline  Desipramine Imipramine  Doxepin  Maprotiline  Nortriptyline  Clomipramine  *SARIs*  Trazodone  *MAOIs*  Isocarboxazid Phenelzine Tranylcypromine Desveniafaxine  *TeCAs*  Mirtazapine | Not reported | Not reported | Not reported | The most commonly prescribed type was SSRIs (N=42,263, 78.1%), then followed by SNRIs, N=14,155, 26.2%), and SARIs ( N=9,592, 17.7%) |
| McIntyre et al.,  2017 | USA | A Secondary data analysis study | Adults | Multiple settings including public and private hospitals, outpatient clinics and emergency departments | 130,626 | A primary diagnosis | Not reported | Not reported | *SSRIs*  No details | Not reported | Not reported | Not reported | Individuals with MDD-MF had statistical significantly (p <0.001) greater medication use during the one-year follow-up period and also greater use of polypharmacy. |
| Massamba et al.,  2017 | Canada | A longitudinal study | Elderly | GP clinics | 263 | A primary diagnosis | GP/physicians | Not reported | *SSRIs*  Citalopram Escitalopram Fluoxetine Fluvoxamine Paroxetine Sertraline  *TCAs*  Desipramine  Nortriptyline  *TeCAs*  Mirtazapine | Not reported | Not reported | Not reported | No specific comments |
| Chon et al.,  2017 | Korea | Review of medical records | Children/ Adolescents | Not reported | 2,190 | A primary diagnosis | Not reported  assumed to be psychiatrists | Not reported | *SSRIs*  Citalopram Escitalopram Fluoxetine Fluvoxamine Paroxetine Sertraline  *SNRIs*  Duloxetine Venlafaxine  *TCAs*  Desipramine  Nortriptyline  *SARIs*  Trazodone  *TeCAs*  Mirtazapine | 91.6% monotherapy, 2 or more types 8.4%, 3 or more 0.6%, most common combination was SSRI and TCA | Not reported | Not reported | No specific comments |
| Bose et al.,  2017 | India | A cross-sectional study | Adults | Private hospital | 200 | A primary diagnosis | Psychiatrists | Not reported | *SSRIs*  Escitalopram Fluoxetine  *TCAs*  Amitriptyline | Not reported | Not reported | Not reported | No specific comments |
| Dharni et al.,  2018 | Australia | Review of medical records | Children/Adolescents | Community care | 189 | A primary diagnosis | Psychiatrist; GP/physicians | Not reported | *SSRIs*  No details | Not reported | Not reported | Not reported | No specific comments |
| Zhong et al,  2018 | 10 Asian/region countries including China, Hong Kong, India, Indonesia, Japan, South Korea, Malaysia, Singapore, Taiwan, and Thailand. | A Secondary data analysis study | Elderly | Public and private hospitals and outpatient clinics/centres | 671 | A primary diagnosis | Psychiatrist | Not reported | *SSRIs*  No details  *SNRIs*  No details  *NASSAs*  No details  *TCA*  No details | Not reported | Not reported | Not reported | Only the broad type of ADs was described. SSRIs were the most commonly prescribed type (88.4%), followed by NaSSA (24.3%) and SNRIs (23.1%). |
| Gers et al.,  2018 | Belgium | Observational case series study | Elderly | Multiple settings | 239 | A primary diagnosis | Psychiatrist; GP/physicians; Other: Geriatricians and Neurologist | Not reported | *SSRIs*  Citalopram Escitalopram Paroxetine Sertraline  *SNRIs*  Duloxetine Venlafaxine  *TCAs*  Amitriptyline  Melitracen  *TeCAs*  Mirtazapine | Not reported | Not reported | Not reported | Incorrect indication for prescribing AD in 25.4% among patients had AD at intake. |
| FataNahas et al.,  2018 | Malaysia | Observational case series study | Adults | Public hospital | 119 | A primary diagnosis | Psychiatrist | Not reported | *SSRIs*  No details  *SNRIs*  No details  *TCAs*  No details  *MAOIs*  No details  *TeCAs*  No details | Not reported | Not reported | Not reported | Only broad drug types were reported, no breakdown. SSRIs comprised the most frequent antidepressants currently being consumed (72.9%), followed by TCAs, whereas MAOIs were the least frequent antidepressants |
| Bandoli et al.,  2018 | USA | A Secondary data analysis study | Pregnant women | Public and private hospitals | 162 | A primary diagnosis | Not reported | Not reported | *SSRIs*  No details  *NASSA*  No details  *TCA*  No details  *MAOIs*  No details | Not reported | Not reported | Not reported | The most common class of antidepressant prescribed was SSRIs (n= 78.3%), followed by bupropion (13.9%), TCAs (n= 8.4%), and other antide-pressants (7.8%) Some women taking more than one class of antidepressant. |
| Verhaak et al.,  2019 | the Netherlands | A Secondary data analysis study | Adults | GP clinics | 326,025 | A primary diagnosis | GP/physicians | Not reported | *SSRIs*  No details  *TCAs*  No details | Not reported | Not reported | Not reported | Of the antidepressants prescribed, SSRIs and TCAs accounted for 52% and 28%, respectively. 60% of prescriptions were for females. 30% of antidepressants were prescribed to those aged 18-44 years, 45% to 45-64 years old and 25% to those above 65 year |
| Vadiei et al.,  2019 | USA | A Secondary data analysis study | Adults | Not reported | 262 | a comorbidity | GP/physicians | Not reported | *SSRIs*  No details  *NASSA*  No details  *TeCAs*  No details | Not reported | Not reported | Not reported | No details on actual medications. SSRIs were the most prescribed class, accounting for approximately 34.59% of antidepressant use). The miscellaneous category (bupropion and mirtazapine) was the second most prescribed class, accounting for 7.25% of antidepressant use. |
| Saito et al.,  2019 | Japan | A cross-sectional study | Children/Adolescents | Multiple settings | 6,080 | Not reported | Psychiatrist; GP/physicians | 322 | *SSRIs*  Escitalopram  Fluvoxamine Paroxetine Sertraline  *SNRIs*  Duloxetine  *TCAs*  No details  *TeCAs*  Mirtazapine | Not reported | Not reported | Less side effect  Familiar with the product  Long history of clinical use and safety profile known  Convenient dosing and/or administration  Effectively relieves symptoms  Low incidence of withdrawal symptoms | No specific comments |
| Tayem et al.,  2020 | Bahrain | A cross-sectional study | All ages | Psychiatric Hospital | 226 | A primary diagnosis | Psychiatrist | Not reported | *SSRIs*  No details  *SNRIs*  No details  *TCAs*  No details | Not reported | Not reported | Not reported | Only broad type of ADs was described. SSIRs was the most commonly prescribed AD of all ADs (41.6%) |
| Lunghi et al.,  2020 | Italy | Retrospective cohort study | All ages | Not reported | 18,307 | Not reported | Psychiatrist; GP/physicians | Not reported | *SSRIs*  Citalopram Escitalopram Fluoxetine Paroxetine Sertraline  *SNRIs*  Duloxetine Venlafaxine  *TCAs*  Amitriptyline  *SARIs*  Trazodone  *TeCAs*  Mirtazapine | Not reported | Not reported | Not reported | No specific comments |
| Lukmanji et al.,  2020 | Canada | A Secondary data analysis study | Children/Adolescent | Primary care | Not reported | Not reported | GP/physicians | 652 | *SSRIs*  Citalopram Escitalopram Fluoxetine Fluvoxamine Paroxetine Sertraline  *SNRIs*  Desvenlafaxine  Duloxetine Venlafaxine  *TCAs*  Amitriptyline  Desipramine Imipramine  Doxepin  Maprotiline  Nortriptyline  Clomipramine  *SARIs*  Trazodone  *TeCAs*  Mirtazapine | Not reported | Not reported | Not reported | No specific comments |
| Heald et al.,  2020 | UK | A Secondary data analysis study | All ages | GP clinics | Not reported | Not reported | GP/physicians | Not reported | Not reported | Not reported | Not reported | Not reported | The main focus of the study was on the change of prescriptions between two time periods, rather than the actual prescribing behaviour |
| Hadia et al.,  2021 | India | Observational case series study | Adults | Public hospital | 37 | A primary diagnosis | Psychiatrist | Not reported | *SSRIs*  Citalopram Escitalopram Fluoxetine  Paroxetine Sertraline  *SNRIs*  Desvenlafaxine  Duloxetine  *TCAs*  Imipramine  *SARIs*  Trazodone  *TeCAs*  Mirtazapine | 52% monotherapy 48% were prescribed 2 or more. | Not reported | Not reported | No specific comments |
| Chen et al.,  2021 | Taiwan | A longitudinal study | Adults | Public hospital | 105 | A primary diagnosis | Psychiatrist | Not reported | Not reported | mean 1.7 (0.9) | Not reported | Not reported | The main focus of the study was on when medications were prescribed not which medication. |
| O'Neill et al.,  2021 | Ireland | A longitudinal study | Elderly | Patients were participants of The Irish Longitudinal Study on Ageing (TILDA) | 817 | Not mentioned but patients were assessed for depressive symptoms using CESDS | Not reported | Not reported | Not reported | Not reported | Not reported | Not reported | No information on the types of AD or any breakdowns. Females were more likely to be long term users than males (OR=1.88, 95%C.I.=1.46-2.46). Primary educated persons were more likely to be long term users than tertiary educated persons (OR=2.02, 95%C.I.=1.55-2.64). |
| Mössinger et al.,  2021 | Germany | A Secondary data analysis study | Adults | GP clinics | 138,097 | A primary diagnosis | GP/physicians | 1188 GP practices | *SSRIs*  Citalopram Escitalopram  *TCAs*  Amitriptyline | An average of 1.4 (0.7) for all ages. | Mean Rx length decreased with age with 5.4(10.5), 5.1 (10.4), and 4.9 (10.0) for 18-29, 30-65, and 65+ age groups respectively. | Not reported | No specific comments |
| Kamran et al.,  2021 | Pakistan | Observational case series study | Adults | Outpatient clinics/centre | 302 | A primary diagnosis | Psychiatrist | Not reported | *SSRIs*  Citalopram Escitalopram Fluoxetine Paroxetine Sertraline  *SNRIs*  Duloxetine Venlafaxine  *TCAs*  Amitriptyline  Desipramine Imipramine  Doxepin  Maprotiline  Nortriptyline  Clomipramine  *SARIs*  Trazodone  *TeCAs*  Mirtazapine | Single type of AD 77%, 22.3% two types, and 0.7% 3 types | Not reported | Not reported | No specific comments |
| Hung et al.,  2021 | Taiwan | A longitudinal study | Adults | Not reported | 97 | A primary diagnosis | Not mentioned exactly just clinicians | Not reported | *SSRIs*  Escitalopram Fluoxetine Fluvoxamine Paroxetine Sertraline  *SNRIs*  Duloxetine Venlafaxine  *TCAs*  Imipramine  Doxepin  *SARIs*  Trazodone  *TeCAs*  Mirtazapine | Not reported | Not reported | Not reported | Most common type was SSRIs 59/97 and among these Sertaline was the most prescribed drug. For first-line medication SSPIs was the preferred option 35/97 and followed by SNRIs 24/97. It was also the most common drug of choice for the 2nd prescribed medication. |
| Hattab et al.,  2021 | Palestine | A Secondary data analysis study | Adults | Outpatient clinics/centre | 159 | A primary diagnosis | Psychiatrist | Not reported | *SSRIs*  No details  *TCAs*  No details | Not reported | Not reported | Not reported | For patients with MDD, SSRIs (45.3%), TCAs (38.3%). |
| Hashimoto et al.,  2021 | Japan | Observational case series study | Adults | Public and private hospitals | 1,238 | A primary diagnosis | Psychiatrist | Not reported | *SSRIs*  Escitalopram Sertraline  *SNRIs*  Duloxetine  *SARIs*  Trazodone  *TeCAs*  Mirtazapine | Not reported | Not reported | Not reported | No specific comments |
| Hansen et al.,  2021 | Norway | A Secondary data analysis study | Adults | GP clinics | 49,967 | A primary diagnosis | GP/physicians | Not reported | *SSRIs*  Citalopram Escitalopram Fluoxetine Fluvoxamine Paroxetine Sertraline  *SNRIs*  Desvenlafaxine  Duloxetine  *TCAs*  Amitriptyline  Desipramine Imipramine  Doxepin  Maprotiline  Nortriptyline  *SARIs*  Trazodone  *TeCAs*  Mirtazapine | Not reported | Not reported | Not reported | No specific comments |
